# Assessing medical students’ attitudes, performance, and usage of ChatGPT in Jeddah, Saudi Arabia

**DOI:** 10.3389/frai.2025.1577911

**Published:** 2025-07-14

**Authors:** Dalia Alammari, Elaf Alamari, Rawan Alamri, Raneem Alharbi, Jumana Felimban, Jana Aljohani

**Affiliations:** ^1^Department of Immunology and Pathology, Faculty in Medicine Program, Ibn Sina National College for Medical Studies, Jeddah, Saudi Arabia; ^2^Health Professions Education, Ibn Sina National College for Medical Studies, Jeddah, Saudi Arabia; ^3^Medicine Program, Ibn Sina National College for Medical Studies, Jeddah, Saudi Arabia

**Keywords:** artificial intelligence, ChatGPT, medical education, educational technology, medical students

## Abstract

**Background:**

ChatGPT, an advanced AI language model, has the potential to significantly enhance medical education by supporting clinical decision-making, facilitating knowledge acquisition, and improving learning outcomes. However, there remains a gap in understanding the risks and concerns surrounding the use of ChatGPT, as well as its impact on the quality of medical education in Jeddah, Saudi Arabia. This study investigates the role of ChatGPT in local medical education, offering insights into medical students’ attitudes and performance concerning the integration of AI into the medical curriculum.

**Methods:**

This cross-sectional study was conducted from March to May 2024. It was approved by the institutional review board. An online survey was distributed to medical students in Jeddah to collect data on their awareness of ChatGPT and its impact on their attitudes and performance. The survey, which included 28 items across 2 sections was developed. For data analysis, Statistical Package for Social Sciences (SPSS) software (version 27.0) was used to conduct statistical analysis.

**Results:**

The final sample comprised 420 participants, of whom 84.3% had heard of ChatGPT, while 74.9% had used it prior to the study. The majority of participants were aged 18–21 (50.5%). Higher GPA and academic progression were significantly associated with greater awareness and performance related to ChatGPT. Additionally, privacy concerns, willingness to incorporate ChatGPT into learning and research, and perceptions of its ease of use were significantly correlated, with a *p*-value < 0.05.

**Conclusion:**

The findings indicate a generally positive perception of ChatGPT among medical students, particularly as they progress in their studies. Associations were observed between ChatGPT usage and students’ academic standing and attitudes toward AI. While the ease of use was appreciated, concerns regarding privacy, ethical implications, and data security were also prominent, reflecting global trends. Further longitudinal and experimental research is necessary to better understand the educational implications of ChatGPT and to ensure its responsible integration into medical curricula.

## 1 Introduction

Artificial intelligence (AI) has garnered global recognition for its ability to tackle complex language comprehension and generation tasks in conversational contexts. In addition, it has made significant improvements in various fields recently, including medical education and healthcare (Sallam et al., 2023; Masters, 2019). The use of AI in medical education is more common in Western countries than in developing countries. The difference can be minimized by more infrastructure support from health organizations in developing countries (Kansal et al., 2022; Ahmed et al., 2022).

Chat Generative Pre-trained Transformer (ChatGPT) is one of the advanced AI-based tools developed by openAI. ChatGPT was first accessible to the public in November 2022. It functions as a virtual teaching aid, delivering students with detailed and direct information, which evolves into interactive simulations. Additionally, it can boost student involvement and improve the learning experience (Sallam et al., 2023; Masters, 2019; Lee, 2023). By incorporating ChatGPT into medical education, educators may be able to enhance the student learning experience in both diagnostic and therapeutic decisions while supplying them with the necessary AI abilities to meet the challenging obstacles they will face in the healthcare industry (Sapci and Sapci, 2020). Therefore, every medical student needs to have adequate knowledge of generative language models since it potentially will play an important role in healthcare in the forthcoming years (Eysenbach, 2023).

According to research conducted in Jordan, it demonstrated good reliability, validity, and usefulness in assessing healthcare students’ attitudes toward ChatGPT evaluated by the Technology Acceptance Model Edited to Assess ChatGPT Adoption. The results highlighted the importance of considering risk perceptions, usefulness, ease of use, attitudes toward technology and behavioral factors when choosing ChatGPT as a tool in healthcare education (Weidener and Fischer, 2024). Similarly, research was carried out on 1 million medical students in Korea, exploring the integration of AI in medical education, and assessing its impact on learning outcomes and knowledge acquisition. Studies have shown that ChatGPT has the potential to enhance learning experiences, support clinical decision-making, and improve engagement among medical students (Lee, 2023). Also, a recent study performed on 487 medical students in Germany, Austria, and Switzerland showed that 71.7% anticipated the constructive influence of AI on the field of medicine. Additionally, 74.9% of respondents expressed a willingness to incorporate AI teaching into their medical education (Caratiquit and Caratiquit, 2023).

These studies have highlighted the benefits of chatbots in providing instant access to information, promoting active learning, and improving knowledge retention (Lee, 2023). Nevertheless, the majority of research has been performed in Western countries, and there is a lack of studies specifically examining the attitudes, performance, and usage of ChatGPT among medical students in Saudi Arabia.

This study aims to investigate the attitudes, performance, and usage of ChatGPT among medical students in Jeddah, Saudi Arabia, with a focus on understanding the potential role of AI in medical education.

## 2 Methods

### 2.1 Study design and participants

This cross-sectional study was conducted through an online questionnaire between March and May 2024. The inclusion criteria of participants in this study were Saudi, non-Saudi, male and female medical students in Jeddah. The participants were selected using a non-probability convenience sampling technique. The minimum sample size for this study was around 400 participants and determined based on a single population formula using Epi info with a sample size calculation of 95% and a margin of error of 0.05. The final number of participants who responded to the survey was 420 medical students. The inclusion criteria were Saudi, non-Saudi male and female medical students who had heard of ChatGPT before. Students (1) who had not heard about ChatGPT, (2) were not studying the medicine program and (3) all participants who filled in the questionnaires incompletely were excluded from the study. Participation in this study was voluntary and recruitment was carried out through official social media, with the questionnaire link being circulated via Twitter, Telegram, and WhatsApp groups.

### 2.2 Data collection method

An online questionnaire was designed using Google Forms. This questionnaire was adapted from a previously validated and reliable tool reported in prior studies (Sallam et al., 2023; Caratiquit and Caratiquit, 2023). Only minor linguistic and contextual adjustments were made to enhance clarity and ensure suitability to the target population and study objectives. These modifications were minimal and did not alter the core constructs or overall structure of the original instrument. The questions were in English and Arabic and aimed to assess medical students’ attitudes, performance, and usage of ChatGPT. The questionnaire consisted of 28 items in two sections. The first section sought socio-demographic information concerning the participants. The second section used 5 items to evaluate attitudes among respondents who had heard of ChatGPT before the study. An additional 13 items evaluated the performance and usage of ChatGPT among respondents who used ChatGPT before the study. The survey was introduced with full explanation of the purpose of the study, and the estimated completion time, and provided an informed consent form for participation. The introductory section explicitly stated that participant anonymity and privacy were guaranteed by refraining from requesting any personal information such as names or contact numbers. This was followed by items assessing gender, age, nationality, GPA, university type (public vs. private), university name, and year of medical education. Then, a single item followed (“Have you heard of ChatGPT before the study?”) with a “yes” response required to move into the next item, while an answer of “no” resulted in a survey submission. The next item was “Have you used ChatGPT before the study?” with “Yes” resulting in the presentation of the full 18 items. An answer of “no” results in the presentation of the first 5 items. There were no duplicates since each respondent was only allowed to complete the questionnaire once by activating the limit to one response option in the settings list, which allows you to only answer the survey via email once. Data from the online questionnaire was automatically collected and stored in an Excel spreadsheet.

### 2.3 Data analysis

The Statistical Package for Social Sciences (SPSS) software (version 27.0) was used to conduct statistical analyses. Descriptive statistics were employed to characterize socio-demographic characteristics as frequencies and percentages, while numerical variables were provided as mean ± standard deviation. We examined the statistical association between categorical variables using the chi-square test and t-test, and the *p*-value was considered statistically significant at *p* < 0.05.

The total scores for the participants were found to follow a normal distribution, as indicated by the Kolmogorov–Smirnov test (*p* = 0.200); therefore, it was decided to use parametric tests such as the students’ t-test and ANOVA for comparison of total scores between different variables. Independent variables included demographic factors (age, gender, GPA), while dependent variables assessed attitudes, performance, and usage of ChatGPT. Confounding variables (e.g., academic year) were controlled. Confidence levels were set at 95% with corresponding confidence intervals reported for each key finding. No missing data were reported. Data were divided into two groups: students who had heard of ChatGPT (*n* = 354) and students who did not hear of ChatGPT (*n* = 66). The participants who had heard of ChatGPT (*n* = 354) were divided into two subgroups: students who had used ChatGPT before the study (*n* = 265) and students who did not use ChatGPT before this study (89). Then the data were sub-grouped by academic year (1st to 6th year and internship) and GPA ranges (on both 4-point and 5-point scales). Quantitative variables such as age were categorized into ranges (e.g., 18–21, 22–25, 26–29).

### 2.4 Risk of bias assessment

Selection bias was minimized by distributing the survey through multiple platforms to ensure diverse participation. Information bias was reduced by using validated and structured questionnaires. Additionally, recall bias was addressed by limiting questions to recent and measurable experiences.

Given the coexistence of 4-point and 5-point GPA systems among participating institutions, all self-reported GPAs were standardized to a common 100-point scale prior to statistical analysis. This step ensured comparability across participants and reduced the risk of analytical bias due to inconsistent grading metrics.

### 2.5 Ethical considerations

The protocol was approved by the Institutional Research Review Board at Ibn Sina National College for Medical Studies, Jeddah, Saudi Arabia (IRRB Number: IRRB-ER/01–23012025). By clicking the survey link, participants provided informed consent before completing the questionnaire. Those who declined were given the option not to participate. Participants were informed of the study’s objectives and were guaranteed anonymity during the survey, protecting the privacy of their data.

## 3 Results

### 3.1 Study participants

**Figure 1:**
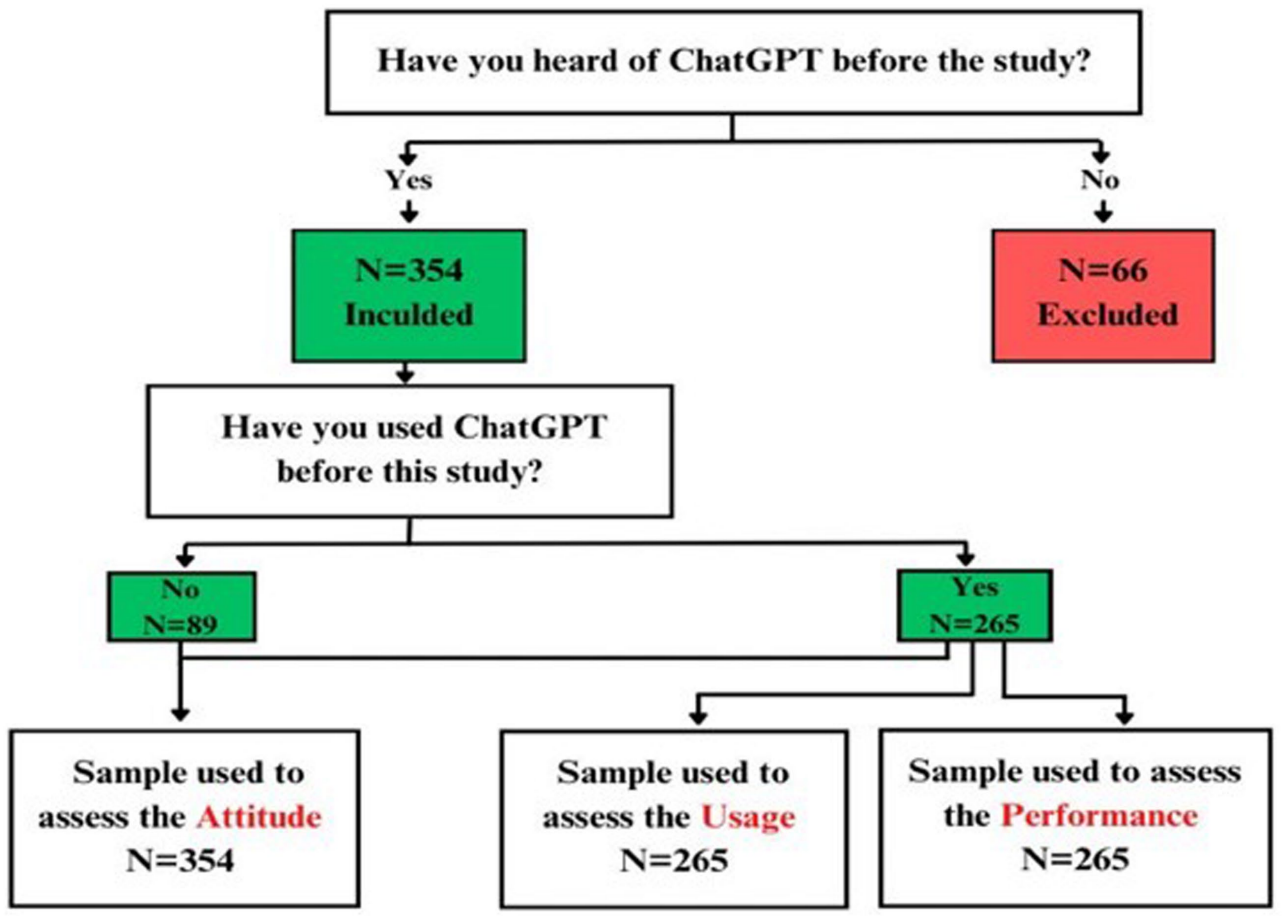
Illustration of the participant selection process for the study.

A total of 420 responses were received. Figure 1 illustrates the participant selection process for the study. Initially, participants were asked if they had heard of ChatGPT before the study. A total of 354 participants (84%) who responded “Yes” were included, while 66 participants (16%) who responded “No” were excluded. Among those included, participants were further asked if they had used ChatGPT before the study. Of these, 89 (25.1%) participants responded “No” and were used to assess attitudes. The 265 (74.9%) participants who responded “Yes” were used to assess attitudes, Usage, and Performance.

Sociodemographic characteristics are shown in Table 1. Females comprised 77.1% (*n* = 324) of the respondents, while males were 22.9% (*n* = 96). The majority were aged 18–21 (50.5%, *n* = 212), followed by those aged 22–25 (41.2%, *n* = 173), 26–29 (7.6%, *n* = 32), and over 30 (0.7%, 3). Most participants were Saudi nationals 85.0% (*n* = 357), with non-Saudis making up 15.0% (*n* = 63). A significant proportion attended private colleges at 87.4% (*n* = 367), while the rest attended public colleges at 12.6% (*n* = 53). Regarding academic performance, for those using a 4-point GPA scale (*n* = 42), most had a GPA between 3.6 and 4 (61.9%, *n* = 26). For those using a 5-point scale (*n* = 378), the largest groups had GPAs between 4.6 and 5 (34.4%, *n* = 130) and 4.1 to 4.5 (30.2%, *n* = 114). Students were at different stages in their academic years, with 20.5% (*n* = 86) in their first year and others spread across subsequent years up to the internship year 4.3% (*n* = 18). A significant majority 84.3% (*n* = 354) had heard of ChatGPT before the study, and 74.9% (*n* = 265) had used it.

**Table 1:** Socio-demographic data among medical students in Jeddah, Saudi Arabia.

Variable			*N*	%
Gender		Male	96	22.9
		Female	324	77.1
Age (Years)		18–21	212	50.5
		22–25	173	41.2
		26–29	32	7.6
		More than 30	3	0.7
Nationality		Saudi	357	85.0
		Non-Saudi	63	15.0
Medical College Type		Private college	367	87.4
		Public college	53	12.6
GPA	Out of 4 (*n* = 42)	Less than 1.5	2	4.8
		1.5–2	1	2.4
		2.1–2.5	1	2.4
		2.6–3	5	11.9
		3.1–3.5	7	16.7
		3.6–4	26	61.9
	Out of 5 (*n* = 378)	Less than 1.5	4	1.1
		1.5–2	4	1.1
		2.1–2.5	7	1.9
		2.6–3	36	9.5
		3.1–3.5	29	7.7
		3.6–4	54	14.3
		4.1–4.5	114	30.2
		4.6–5	130	34.4
Academic year		1st year	86	20.5
		2nd year	66	15.7
		3rd year	66	15.7
		4th year	121	28.8
		5th year	40	9.5
		6th year	23	5.5
		Internship year	18	4.3
Have you heard of ChatGPT before this study?		No	66	15.7
		Yes	354	84.3
Have you used ChatGPT before this study?		No	89	25.1
		Yes	265	74.9

### 3.2 Prior knowledge and usage of ChatGPT among the study participants

The association between the study variables and previous knowledge of ChatGPT. Gender showed a marginal association with awareness, with males being slightly more aware than females (*p* = 0.052). Age did not significantly affect awareness (*p* = 0.205). About GPA, when measured on a 4-point scale, it showed no significant association (*p* = 0.652), but on a 5-point scale, a higher GPA was significantly associated with greater awareness (*p* < 0.001). Lastly, the academic year correlated significantly with awareness, indicating that awareness increased with progression through the academic years (*p* = 0.001) in Table 2.

**Table 2:** Comparison between students who have heard about ChatGPT and those who have not heard about it before as regards socio-demographic data (*n* = 354).

Variable			Heard of ChatGPT before		*p*-value
			Yes	No	
Gender		Male	87 (90.6%)	9 (9.4%)	0.052
		Female	267 (82.4%)	57 (17.6%)	
Age (years)		18–21	171 (80.7%)	41 (19.3%)	0.205
		22–25	152 (87.9%)	21 (12.1%)	
		26–29	28 (87.5%)	4 (12.5%)	
		Above 30	3 (100%)	0 (0%)	
Nationality		Saudi	295 (82.6%)	62 (17.4%)	0.027^*^
		Non-Saudi	59 (93.7%)	4 (6.3%)	
Medical College Type		Private	304 (82.8%)	63 (17.2%)	0.031^*^
		Public	50 (94.3%)	3 (5.7%)	
GPA	Out of 4	< 1.5	1 (50%)	1 (50%)	0.652
		1.5–2	1 (100%)	0 (0.0%)	
		2.1–2.5	1 (100%)	0 (0.0%)	
		2.6–3	2 (40%)	3 (60%)	
		3.1–3.5	6 (85.7%)	1 (14.3%)	
		3.6–4	20 (75%)	6 (23.1%)	
	Out of 5	< 1.5	2 (50%)	2 (50%)	<0.001^*^
		1.5–2	0 (0.0%)	4 (100%)	
		2.1–2.5	7 (100%)	0 (0.0%)	
		2.6–3	24 (66.7%)	12 (33.3%)	
		3.1–3.5	27 (93.1%)	2 (6.9%)	
		3.6–4	42 (77.8%)	12 (22.2%)	
		4.1–4.5	100 (87.7%)	14 (12.3%)	
		4.6–5	121 (93.1%)	9 (6.9%)	
Academic year		1st year	66 (76.7%)	20 (23.3%)	0.001^*^
		2nd year	47 (71.2%)	19 (28.8%)	
		3rd year	54 (81.8%)	12 (18.2%)	
		4th year	113 (93.4%)	8 (6.6%)	
		5th year	35 (87.5%)	5 (12.5%)	
		6th year	22 (95.7%)	1 (4.3%)	
		Internship	17 (94.4%)	1 (5.6%)	

Based on the Chi-square test, the level of significance at *p*-value<0.05. *p-value <0.05 is considered statistically significant.

The association between the study variables and previous usage of ChatGPT is represented in Table 3. The students’ gender showed a significant association with usage, where a higher percentage of males reported using ChatGPT compared to females (86.2% vs. 71.2%, *p* = 0.005). However, age did not significantly affect usage (*p* = 0.585). Among students who had a 4-point GPA, no significant association was found with ChatGPT usage (*p* = 0.695). However, it was found that among students who had a GPA of 5, students who had a GPA of 4.1–4.5 and 4.6–5 had significantly higher usage compared to others (*p* = 0.018). Lastly, academic year was significantly associated with the usage, with higher usage reported in later academic years (*p* < 0.001).

**Table 3:** Comparison between students who have used ChatGPT and those who have not used it before as regards socio-demographic data (*n* = 265).

Variable			Used ChatGPT before		*p-*value
			Yes	No	
Gender		Male	75 (86.2%)	12 (13.8%)	0.005^*^
		Female	190 (71.2%)	77 (28.8%)	
Age (years)		18–21	127 (35.9%)	44 (25.7%)	0.585
		22–25	116 (76.3%)	36 (23.7%)	
		26–29	19 (67.9%)	9 (32.1%)	
		Above 30	3 (100%)	0 (0%)	
Nationality		Saudi	221 (74.9%)	74 (25.1%)	0.956
		Non-Saudi	44 (74.6%)	15 (25.4%)	
Medical College Type		Private	220 (62.1%)	84 (27.6%)	0.008^*^
		Public	45 (90%)	5 (10%)	
GPA	out of 4	< 1.5	1 (100%)	0 (0%)	0.695
		1.5–2	1 (100%)	0 (0%)	
		2.1–2.5	1 (100%)	0 (0%)	
		2.6–3	1 (50%)	1 (50%)	
		3.1–3.5	5 (83.3%)	1 (16.7%)	
		3.6–4	17 (85%)	3 (15%)	
	out of 5	< 1.5	1 (50%)	1 (50%)	0.018^*^
		1.5–2	0 (0%)	0 (0%)	
		2.1–2.5	2 (28.6%)	5 (71.4%)	
		2.6–3	14 (58.3%)	10 (41.7%)	
		3.1–3.5	23 (85.2%)	4 (14.8%)	
		3.6–4	29 (69%)	13 (31%)	
		4.1–4.5	80 (80%)	20 (20%)	
		4.6–5	90 (74.4%)	31 (25.6%)	
Academic year		1st year	42 (63.6%)	24 (36.4%)	<0.001^*^
		2nd year	35 (74.5%)	12 (25.5%)	
		3rd year	32 (59.3%)	22 (40.7%)	
		4th year	98 (86.7%)	15 (13.3%)	
		5th year	29 (82.9%)	6 (17.1%)	
		6th year	19 (86.4%)	3 (13.6%)	
		Internship	10 (58.8%)	7 (41.2%)	

Based on the Chi-square test, the level of significance at *p*-value<0.05. *P-value < 0.05 is considered statistically significant.

**Figure 2:**
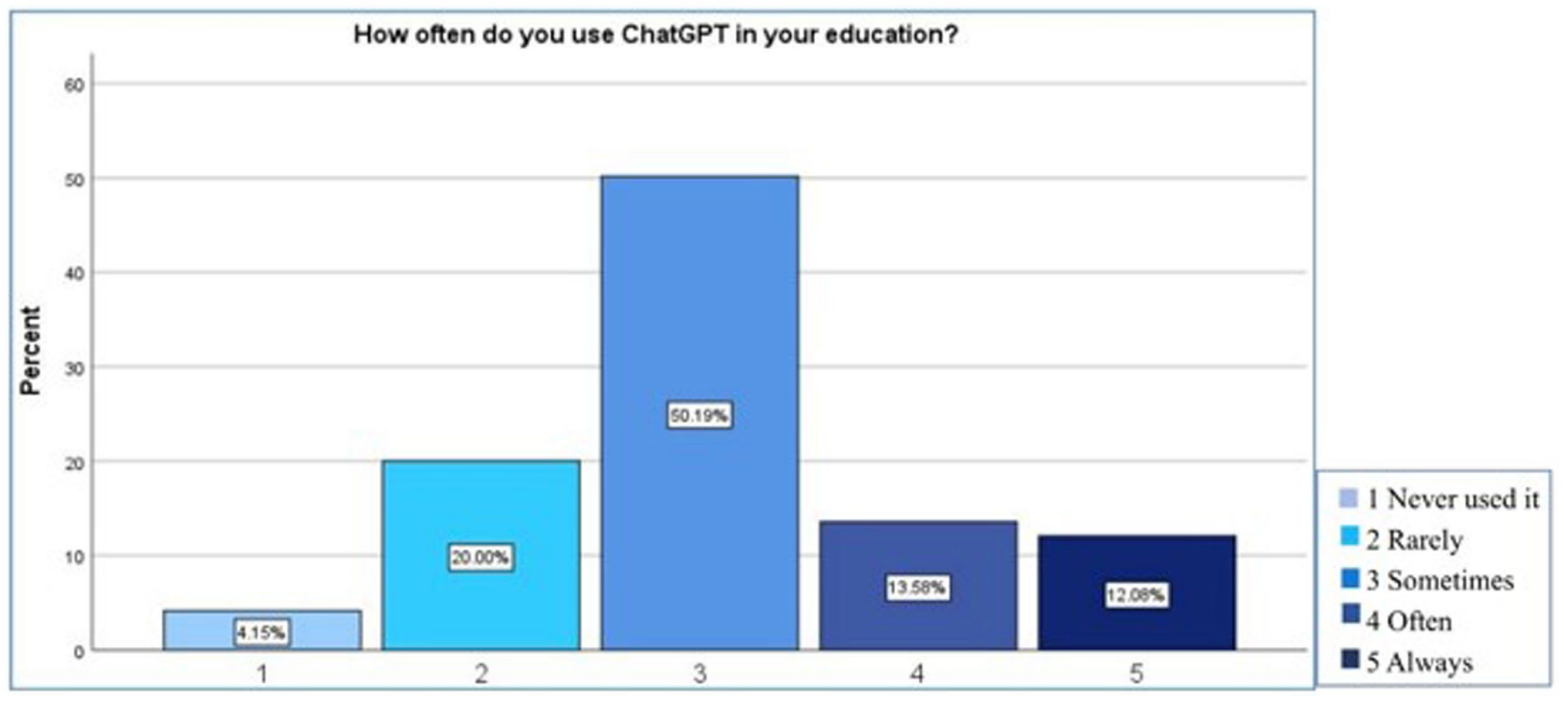
Integration and usage of ChatGPT in education among studied participants.

When the integration and use of ChatGPT in education was assessed in Figure 2 among 265 respondents, it was found that only a few 4.2% (*n* = 11) never used it, 20% (*n* = 53) used it rarely, 50.2% (*n* = 133) used it sometimes, 13.6% (*n* = 36) used it often and 12.1% (*n* = 32) used it always.

### 3.3 Factorability of the correlation matrix of the attitude scale

A comparison between attitudes among respondents who had heard of ChatGPT only and those who had heard and used it among medical students is shown in Table 4.

**Table 4:** Comparison of attitudes between those who have heard of ChatGPT but not used it and those who have heard of ChatGPT and used it among medical students in Jeddah, Saudi Arabia, (*n* = 354).

Variable	Heard of ChatGPT but have not used it	Heard of ChatGPT and used it	t value (students ‘t’ test)	*p*-value
	Mean (SD)	Mean (SD)		
I am concerned that using ChatGPT would lead to accusations of plagiarism	2.80 (1.13)	2.91 (1.16)	−0.779	0.436
I am concerned about the potential privacy risks associated with using ChatGPT	3.09 (1.04)	2.77 (1.16)	2.309	0.021^*^
Who is afraid of becoming too dependent on technology like ChatGPT and not developing critical thinking	3.26 (1.32)	3.48 (1.21)	−1.450	0.148
Who is willing to use technologies such as ChatGPT for learning and researching	3.48 (1.11)	3.92 (1.05)	−3.3712	<0.001^*^
Who believes that technology like ChatGPT is easy and quick to use for learning	3.76 (1.04)	4.03 (1.10)	−2.030	0.043^*^

Based on the independent t-test, the level of significance at *p* value<0.05. *P-value < 0.05 is considered statistically significant.

Concerns about potential privacy risks associated with ChatGPT were significantly higher among those who had heard of it but had not used it, compared to users (mean difference = 0.32, *t* = 2.309, *p* = 0.021). Users also showed a significantly greater willingness to use technologies like ChatGPT for learning and research (mean difference = 0.44, *t* = −3.3712, *p* < 0.001). Additionally, users perceived technology like ChatGPT as easier and quicker to learn than non-users (mean difference = 0.27, *t* = −2.030, *p* = 0.043). However, concerns about accusations of plagiarism and fear of dependence on technology did not significantly differ between the two groups (*p* > 0.05 for both).

### 3.4 Factorability of the correlation matrix of the performance scale

A comparison between the performance and outcomes among medical students in Jeddah, Saudi Arabia with the sociodemographic data of respondents is represented in Table 5. Age showed significant differences in mean overall performance where students aged 26–29 years (3.48 ± 0.89), and over 30 years (4.07 ± 0.12) showed higher mean performance scores (*p* = 0.028) compared to younger age groups. Gender, nationality, college type, GPA, and academic year did not show significant differences (*p* > 0.05).

**Table 5:** Comparison of mean overall score of performance with sociodemographic characteristics, (*n* = 265).

Variable			Mean	SD	*p*-value
Gender		Male	3.16	0.81	0.204
		Female	3.00	0.94	
Age (Years)		18–21	2.98	0.98	0.028^*^
		22–25	3.02	0.80	
		26–29	3.48	0.89	
		> 30	4.07	0.12	
Nationality		Saudi	3.08	0.92	0.181
		Non-Saudi	2.88	0.81	
Medical College type		Private college	3.03	0.90	0.590
		Public college	3.11	0.93	
GPA	Out of 4	< 1.5	3.00	.	0.575
		1.5–2	2.20	.	
		2.1–2.5	3.80	.	
		2.6–3	2.80	.	
		3.1–3.5	3.08	0.23	
		3.6–4	2.75	1.12	
	Out of 5	< 1.5	3.40	.	0.564
		2.1–2.5	2.40	1.13	
		2.6–3	3.13	1.22	
		3.1–3.5	3.29	0.90	
		3.6–4	3.23	0.98	
		4.1–4.5	3.11	0.84	
		4.6–5	2.96	0.90	
Academic Year		1st year	2.94	0.98	0.680
		2nd year	3.23	1.10	
		3rd year	3.09	1.03	
		4th year	3.07	0.76	
		5th year	2.88	0.85	
		6th year	2.91	0.88	
		Internship year	3.24	1.02	

Based on independent t-test and one-way ANOVA, the level of significance at *p*-value<0.05. *P-value < 0.05 is considered statistically significant.

### 3.5 Factorability of the correlation matrix of the usage scale

A comparison between the usage of ChatGPT and the sociodemographic data of the respondents is shown in Table 6. Age showed significant differences where students aged 26–29 years (3.86 ± 0.57), and over 30 years (4.14 ± 0.52) showed higher mean usage scores compared to younger age groups (*p* = 0.032). At the same time, gender, nationality, college type, GPA, and academic year did not show significant differences (*p* > 0.05).

**Table 6:** Comparison of mean overall score of ChatGPT usage with sociodemographic characteristics (*n* = 265).

Variable			Mean	SD	*p*-value
Gender		Male	3.58	0.66	0.110
		Female	3.41	0.81	
Age (years)		18–21	3.46	0.80	0.032^*^
		22–25	3.38	0.74	
		26–29	3.86	0.57	
		> 30	4.14	0.52	
Nationality		Saudi	3.48	0.77	0.404
		Non-Saudi	3.37	0.78	
Medical College Type		Private college	3.46	0.77	0.964
		Public college	3.45	0.80	
GPA	Out of 4	< 1.5	3.00	.	0.172
		1.5–2	4.57	.	
		2.1–2.5	3.86	.	
		2.6–3	3.29	.	
		3.1–3.5	3.63	0.94	
		3.6–4	3.25	0.77	
	Out of 5	< 1.5	1.00	.	0.123
		2.1–2.5	3.64	0.71	
		2.6–3	3.43	0.93	
		3.1–3.5	3.46	0.80	
		3.6–4	3.72	0.78	
		4.1–4.5	3.50	0.64	
		4.6–5	3.43	0.76	
Academic year		1st year	3.38	0.78	0.430
		2nd year	3.71	0.85	
		3rd year	3.55	0.81	
		4th year	3.40	0.69	
		5th year	3.40	0.86	
		6th year	3.33	0.56	
		Internship year	3.51	1.06	

Based on independent t-test and one-way ANOVA, the level of significance at *p*-value<0.05. *P-value < 0.05 is considered statistically significant.

## 4 Discussion

As the world approaches modernization and globalization, the digitalization of various fields has become a common phenomenon in this contemporary era. A broad range of medical students currently utilize AI tools in their daily academic tasks. Therefore, this study aimed to evaluate medical students’ attitudes regarding ChatGPT use in their education, examine their performance, and investigate the integration and use of ChatGPT.

Limited research currently exists on medical students’ attitudes, performance, and usage of ChatGPT in Jeddah, Saudi Arabia. This research could provide insight into how students perceive and interact with this emerging technology within their educational environment and enable a better understanding of the potential benefits and challenges of integrating AI tools like ChatGPT into medical education. According to previous studies, the usage of ChatGPT ranges from 11 to 85% globally. Collectively, the adoption of ChatGPT among university students is increasing yearly due to society becoming significantly dependent on technology for communicating, learning, and problem-solving (Sallam et al., 2024).

Our study revealed that more male medical students were familiar with ChatGPT, a trend also observed in a study from Jordan. Which demonstrated strong consistency, accuracy and practical value in assessing healthcare students’ attitudes toward ChatGPT, using an adapted Technology Acceptance Model specifically designed for evaluating ChatGPT adoption. This might point to some underlying gender differences in how students engage with technology, as it seems that males are often more inclined to explore and interact with AI tools (Sallam et al., 2023).

We also found that older medical students were more likely to know about ChatGPT, which aligns with the Jordanian findings and could reflect their increased exposure to various academic tools and technologies over the years. Interestingly, students in their final years were particularly knowledgeable about ChatGPT, mirroring the trends observed in the Jordanian study. This could be because, as they near the end of their training, they tend to use more advanced academic resources (Sallam et al., 2023). However, it is important to note that age was not a statistically significant factor in ChatGPT awareness, despite its association with usage. This distinction highlights the need for caution when attributing increased familiarity solely to age.

Our study identified a significant gender-based difference in ChatGPT usage, with male students reporting higher usage rates than females. This aligns with a study conducted in Jordan, which also indicated that male students are more likely to use ChatGPT than their female counterparts. Interestingly, while our research did not find age to be a significant factor in ChatGPT usage, the Jordanian study noted a positive correlation between age and usage, suggesting that older students tend to use the tool more often. This could be attributed to their greater comfort with technology, improved time management skills, and increased confidence in assessing AI-generated content. Additionally, we observed that ChatGPT usage tends to rise as medical students progress through their academic years, a trend mirrored in the Jordanian study. This likely reflects a growing familiarity with technology and an increasing need to meet academic demands as students advance in their education. Overall, our findings highlight a positive relationship between academic progression and the adoption of AI tools (Sallam et al., 2023).

We found no significant relationship between GPA (on a 4-point scale) and ChatGPT usage concerning academic performance. However, a study conducted in the UAE revealed that students with lower GPAs tend to use ChatGPT more frequently as a supportive learning tool. From both studies it can be said that while ChatGPT might not have a direct effect on the academic outcomes of higher-achieving students, it could be an essential resource for students encountering academic challenges (Sallam et al., 2024).

Conversely, our research found a significant correlation between GPA (on a 5-point scale) and the use of ChatGPT, particularly among students with GPAs ranging from 3.1 to 3.5. This finding aligns with a multinational Arab study, which demonstrated that greater engagement with educational resources is associated with improved academic performance. Together, these findings emphasize the importance of encouraging students to utilize ChatGPT effectively, as it has the potential to enhance overall academic performance, especially for those in the middle GPA range (Masters, 2019).

In our research exploring medical students’ attitudes toward ChatGPT in Jeddah, three key points were significant regarding students who had already heard of and used ChatGPT compared to international studies: privacy concerns, willingness to incorporate ChatGPT for learning and research, and perceptions of its ease of use. In our study, many students expressed significant concerns about the privacy breach risks associated with using ChatGPT. Similarly, a study conducted in Jordan found that medical students shared similar worries about the potential data privacy risks and flaws of ChatGPT, especially regarding the sharing of sensitive information (Sallam et al., 2023). While privacy concerns were frequently cited by participants, it is likely that such concerns are shaped not only by personal preferences but also by cultural values, gender norms, and institutional ambiguity regarding AI use. The absence of clear guidelines or policies may contribute to uncertainty and hesitation, highlighting the need for a more structured ethical framework for AI integration in medical education. Concerns regarding the inaccuracy, ethical implications, risk of bias, plagiarism, and copyright issues of using AI in medical education are acknowledged worldwide, with researchers cautioning about the ethical consequences of ChatGPT misuse. Students should maintain all necessary precautions such as those related to data privacy, informed consent, and potential bias in decision-making, especially in medical education. Students’ concerns regarding risk barriers negatively influenced their intention to use ChatGPT in medical education. On the other hand, increased familiarity with novel technologies and a low level of perceived risks are observed in non-medical disciplines, especially among students who are routinely exposed to emerging technologies such as those at technology-related colleges (Sallam et al., 2023; Sallam et al., 2024; Giannos, 2023; Abdaljaleel et al., 2024).

Additionally, students who were familiar with ChatGPT were more willing to use it for academic purposes, which is consistent with findings from Jordan’s study, which positively showed that students valued ChatGPT for its perceived usefulness and its ability to quickly retrieve information and improve learning efficiency (Sallam et al., 2023; Alfadda and Mahdi, 2021). The willingness to use ChatGPT also arose from positive experiences with ChatGPT’s ability to simplify complex topics, integrate content sharing, and facilitate access to educational information. Reflecting insights from research on ChatGPT’s performance in medical biochemistry, which highlights AI’s usefulness in enhancing student engagement, learning, and understanding while warning against over-reliance (Sallam et al., 2024; Alfadda and Mahdi, 2021; Surapaneni, 2023; Okonkwo and Ade-Ibijola, 2021).

Furthermore, many students in this research found ChatGPT easy and quick to use, which aligns with different research from different countries, demonstrating that AI tools are easy to adopt without extensive physical effort or a steep learning curve which positively influences perceived usefulness and intention to use (Sallam et al., 2024; Almogren et al., 2024; Na et al., 2022). A study in UAE highlighted that university students appreciated the system’s ability to understand and respond to queries in a simple language format, reducing the need for advanced knowledge and skills to effectively interact with it (Sallam et al., 2024). More studies also emphasize the global availability of ChatGPT and its user-friendly and efficiency in managing large volumes of information as major advantages. However, despite its perceived ease of use, concerns remain about the reliability, reputability, and depth of AI-generated content. Addressing these concerns is crucial to ensure that ChatGPT is used effectively in learning environments while maintaining academic standards and clinical integrity (Sallam et al., 2023; Sallam et al., 2024; Giannos, 2023).

On the other hand, when examining medical students’ attitudes toward ChatGPT in their education. Interestingly, no significant differences between students who had heard of and used ChatGPT and their concerns about plagiarism or dependence on the technology were found. While AI-generated content has the potential to cause plagiarism issues (Jarrah et al., 2023), students showed little worry about it. The relatively low concern about plagiarism may stem from a lack of awareness rather than the absence of risk. This underscores the need for targeted education on ethical AI usage and academic integrity, especially in medical training. This indicates they trust in their capability to use ChatGPT ethically. Nevertheless, this highlights the importance of educational programs in educating students about ethical considerations and possible risks they may not yet fully understand (Sallam, 2023; Borji, 2023). Offering advice on academic honesty can promote the responsible utilization of AI resources.

In our study, the students’ concerns about relying too much on ChatGPT and possibly compromising one’s critical thinking abilities did not differ significantly among the users. This could suggest that ChatGPT is being seen as a tool to assist instead of a dependency, helping current mental processes instead of substituting them. This aligns with research showing that most students in other studies view AI as an ally or a tool instead of a competitor (Bisdas et al., 2021).

These findings suggest that while medical students are open to integrating AI technologies such as ChatGPT, there is a need for curricula to focus on ethical applications and improving skills. Future studies need to investigate how educational systems can promote these goals, ensuring that AI technologies improve learning without compromising academic integrity or critical thinking.

In our research exploring medical students’ usage of ChatGPT and performance in Jeddah. The results indicated a significant relationship between age and the performance and usage of ChatGPT among medical students, with older students (26–29 years and over 30 years) showing higher performance and usage than younger students. This finding aligns with the study by Lee in 2023, which suggested that older students, due to their greater experience with complex medical concepts, are more likely to utilize Al tools like ChatGPT effectively. In both studies, older students recognized the benefits of Al in enhancing their learning outcomes and handling more advanced coursework.

However, in contrast to Weidener and Fischer in 2024 found that age differences in Al adoption among students were not significant in their cross-sectional study across different medical institutions; this study identified a clear age-related pattern in performance and usage. The reason for this difference could be due to the specific cultural and educational context in Jeddah, where older students may have more responsibilities and seek efficient learning tools like ChatGPT to manage their academic workload.

Our study found no significant differences between male and female students in terms of their performance and usage of ChatGPT, indicating that both genders benefit equally from the technology.

This is consistent with the findings of Sallam et al. in 2023, who also reported that gender was not a significant factor in Al adoption among healthcare students in Jordan. Both studies highlighted the gender-neutral nature of Al tools like ChatGPT, which offer accessible and effective learning support to all students, regardless of gender. However, while Lee (2023) suggested that male students in some contexts might show higher adoption rates due to greater familiarity with technology, this study did not find such a pattern. The lack of significant gender differences in this research could be attributed to the growing familiarity with digital tools among both male and female students in Saudi Arabia, reflecting broader trends in technological adoption in the region.

The results of this research showed no significant correlation between the students’ GPA and the performance or usage of ChatGPT. This mirrors the findings of Weidener and Fischer in 2024, who reported similar results, suggesting that AI tools like ChatGPT provide benefits that are independent of the student’s prior academic standing. In both studies, students across different GPA ranges found ChatGPT useful in enhancing their understanding of medical content, indicating that the tool is equally accessible and beneficial for students with varying academic abilities.

In contrast, in 2023, Caratiquit and Caratiquit suggested that students with higher motivation levels (often correlated with higher GPAs) might utilize AI tools more effectively; this study did not find GPA to be a significant factor. This could be due to the structured nature of medical education in Saudi Arabia, where students are encouraged to use all available resources, including AI, regardless of their academic performance (Caratiquit and Caratiquit, 2023).

Contrary to some expectations, this study did not find a significant relationship between academic year and performance or usage of ChatGPT, suggesting that students across all years of medical education in Jeddah used the tool similarly. This differs from Lee in 2023, who noted that students in more advanced years of study were more likely to adopt AI tools due to the increasing complexity of their coursework.

The non-significant results in this study could be explained by the widespread availability of ChatGPT across all levels of medical education, making it a valuable resource for students at both early and advanced stages. In Saudi Arabia, medical students might be encouraged to adopt technological tools from the beginning of their studies, which could explain their uniform usage across different academic years (Lee, 2023).

## 5 Limitations

The main limitation of this study is its relatively small sample size, which may be attributed to the complexity and length of the survey instrument—potentially contributing to respondent fatigue and discouraging participation. In addition, the use of a non-probability convenience sampling method, with distribution via various social media platforms, may have introduced the selection bias. This approach likely overrepresented technologically proficient students or those with a particular interest in artificial intelligence, which may limit the generalizability of the findings to the broader population of medical students in Saudi Arabia. Furthermore, the study relied on self-reported data, which may be influenced by social desire bias, particularly in students’ responses regarding AI usage and attitudes. Digital literacy and internet access, though not measured in this study, could have affected both participation and response quality. Additionally, institutional policies regarding AI usage were not accounted for, which may have shaped students’ attitudes and behaviors. From an analytical standpoint, the absence of multivariate statistical methods, such as logistic or linear regression, limits the ability to adjust for potential confounding variables including age, gender, academic year, and GPA. Future studies are encouraged to employ such methods to derive more robust and adjusted insights. Lastly, while the findings of this study may be generalizable to students in urban academic settings with comparable educational structures, differences in access to technology and cultural attitudes may limit their broader applicability. As a cross-sectional study, it also cannot establish causal relationships between ChatGPT use and educational outcomes.

While ChatGPT is accessible and beneficial across various academic and demographic groups, its integration requires ethical guidelines addressing privacy and over-reliance. Future studies should consider employing longitudinal or mixed-method designs to capture the evolving nature of AI integration in medical education. In-depth qualitative research could provide richer insights into students lived experiences with ChatGPT, particularly in culturally diverse settings. Comparative studies across institutions and countries may also help delineate universal trends from context-specific patterns in AI adoption.

### 5.1 Conclusion

Based on the findings of the study, several recommendations are proposed to enhance the ethical and effective integration of ChatGPT in medical education. First, comprehensive guidelines should be developed and implemented to address key issues such as privacy, ethical considerations, data security, and informed consent, thereby alleviating concerns raised by students. Medical education programs are encouraged to incorporate dedicated modules on AI ethics, covering topics such as data privacy, algorithmic bias, and responsible usage. Additionally, institutional policies should be established or clarified to support students in using AI tools both ethically and effectively. Educational workshops and training sessions should be organized regularly to help students understand the appropriate use of AI tools while avoiding over-reliance on them. Continuous assessment of ChatGPT’s impact on learning outcomes and academic integrity is also recommended, with feedback shared between students and faculty to guide improvements. Lastly, faculty should be encouraged to actively support students in balancing AI-assisted learning with traditional methods, ensuring that the use of ChatGPT enhances rather than diminishes the overall educational experience.

## 6 Conclusion

In conclusion, this study highlights both the opportunities and challenges associated with the integration of AI tools like ChatGPT into medical education, with a focus on medical students in Jeddah, Saudi Arabia. The use of ChatGPT was found to be associated with higher GPA and academic progression, and greater familiarity and usage were reported more frequently among male students and those attending private colleges. Attitudes toward ChatGPT appeared to be influenced by factors such as privacy concerns and perceived ease of use, suggesting a balance between the tool’s potential benefits and perceived risks. While many students reported that ChatGPT supported their learning and helped simplify complex topics, notable concerns remained regarding privacy, ethics, over-reliance, and academic integrity. These findings emphasise the need for careful consideration of both the advantages and risks associated with AI adoption in medical education.

## Data Availability

The raw data supporting the conclusions of this article will be made available by the authors, without undue reservation.

## References

[ref1] Abdaljaleel M. Barakat M. Alsanafi M. Salim N. A. Abazid H. Malaeb D.. (2024). A multinational study on the factors influencing university students' attitudes and usage of ChatGPT. Sci. Rep. 14:1983. doi: 10.1038/s41598-024-52549-8, PMID: 38263214 PMC10806219

[ref2] Ahmed Z. Bhinder K. K. Tariq A. Tahir M. J. Mehmood Q. Tabassum M. S.. (2022). Knowledge, attitude, and practice of artificial intelligence among doctors and medical students in Pakistan: a cross-sectional online survey. Ann Med Surg (Lond). 76:103493. doi: 10.1016/j.amsu.2022.103493, PMID: 35308436 PMC8928127

[ref3] Alfadda H. A. Mahdi H. S. (2021). Measuring students' use of zoom application in language course based on the technology acceptance model (TAM). J. Psycholinguist. Res. 50, 883–900. doi: 10.1007/s10936-020-09752-1, PMID: 33398606 PMC7781650

[ref4] Almogren A. S. Al-Rahmi W. M. Dahri N. A. (2024). Exploring factors influencing the acceptance of ChatGPT in higher education: a smart education perspective. Heliyon 10:e31887. doi: 10.1016/j.heliyon.2024.e31887, PMID: 38845866 PMC11154614

[ref5] Bisdas S. Topriceanu C. C. Zakrzewska Z. Irimia A. V. Shakallis L.. (2021). Artificial intelligence in medicine: a multinational multi-center survey on the medical and dental students’ perception. Front. Public Health 9:795284. doi: 10.3389/fpubh.2021.795284, PMID: 35004598 PMC8739771

[ref6] Borji A. (2023). Categorical Archive of ChatGPT Failures. arXiv. Preprint posted online on May 9, doi: 10.21203/rs.3.rs-2895792/v1

[ref7] Caratiquit K. D. Caratiquit L. J. C. (2023). Chat GPT as an academic support tool on the academic performance among students: the mediating role of learning motivation. J. Soc. Humanity Educ. 4, 21–33. doi: 10.35912/jshe.v4i1.1558

[ref8] Eysenbach G. (2023). The role of chat GPT, generative language models, and artificial intelligence in medical education: a conversation with chat GPT and a call for papers. Jmir Med. Educ. 6:e46885. doi: 10.2196/46885 PMC10028514 36863937

[ref9] Giannos P. (2023). Performance of ChatGPT on UK standardized admission tests: insights from the BMAT, TMUA, LNAT, and TSA examinations. Jmir. Med. Educ. 9:e47737. doi: 10.2196/47737, PMID: 37099373 PMC10173042

[ref10] Jarrah A. M. Wardat Y. Fidalgo P. (2023). Using ChatGPT in academic writing is (not) a form of plagiarism: what does the literature say? Online J. Commun. Media Technol. 13:e202346. doi: 10.30935/ojcmt/13572

[ref11] Kansal R. Bawa A. Bansal A. Trehan S. Goyal K. Goyal N.. (2022). Differences in knowledge and perspectives on the usage of artificial intelligence among doctors and medical students of a developing country: a cross-sectional study. Cureus. 14:e21434. doi: 10.7759/cureus.21434, PMID: 35223222 PMC8860704

[ref12] Lee H. (2023). The rise of chat GPT: exploring its potential in medical education. Anat. Sci. Educ. 17, 926–931. doi: 10.1002/ase.2270, PMID: 36916887

[ref13] Masters K. (2019). Artificial intelligence in medical education. Med. Teach. 41, 976–980. doi: 10.1080/0142159X.2019.1595557, PMID: 31007106

[ref14] Na S. Heo S. Han S. Shin Y. Roh Y. (2022). Acceptance model of artificial intelligence (AI)-based technologies in construction firms: applying the technology acceptance model (TAM) in combination with the technology organisation–environment (TOE) framework. Buildings 12:90. doi: 10.3390/buildings12020090

[ref15] Okonkwo C. W. Ade-Ibijola A. (2021). Chatbots applications in education: a systematic review. Comput. Educ. Artif. Intell. 2:100033. doi: 10.1016/j.caeai.2021.100033

[ref16] Sallam M. (2023). ChatGPT utility in healthcare education, research, and practice: systematic review on the promising perspectives and valid concerns. Healthcare 11:887. doi: 10.3390/healthcare11060887, PMID: 36981544 PMC10048148

[ref17] Sallam M. Elsayed W. Al-Shorbagy M. Barakat M. El Khatib S. Ghach W.. (2024). Chat GPT usage and attitudes are driven by perceptions of usefulness, ease of use, risks, and psycho-social impact: a study among university students in the UAE. Front. Educ. 9:1414758. doi: 10.3389/feduc.2024.1414758

[ref18] Sallam M. Salim N. A. Barakat M. Al-Mahzoum K. AlTammemi A. B. Malaeb D.. (2023). Assessing health students' attitudes and usage of chat GPT in Jordan: validation study. Jmir Med. Educ.:e48254. doi: 10.2196/48254 37578934 PMC10509747

[ref19] Sapci A. H. Sapci H. A. (2020). Artificial intelligence education and tools for medical and health informatics students: systematic review. JMIR Med Educ. 6:e19285. doi: 10.2196/19285, PMID: 32602844 PMC7367541

[ref20] Surapaneni K. (2023). Assessing the performance of ChatGPT in medical biochemistry using clinical case vignettes: observational study. JMIR Med. Educ. 9:e47191. doi: 10.2196/47191, PMID: 37934568 PMC10664016

[ref21] Weidener L. Fischer M. (2024). Artificial intelligence in medicine: cross-sectional study among medical students on application, education, and ethical aspects. Jmir Med. Educ. 10:e51247. doi: 10.2196/51247, PMID: 38180787 PMC10799276

